# Replacement of Chalcone-Ethers with Chalcone-Thioethers as Potent and Highly Selective Monoamine Oxidase-B Inhibitors and Their Protein-Ligand Interactions

**DOI:** 10.3390/ph14111148

**Published:** 2021-11-11

**Authors:** Bijo Mathew, Jong Min Oh, Ahmed Khames, Mohamed A. Abdelgawad, T. M. Rangarajan, Lekshmi R. Nath, Clement Agoni, Mahmoud E. S. Soliman, Githa Elizabeth Mathew, Hoon Kim

**Affiliations:** 1 Department of Pharmaceutical Chemistry, Amrita School of Pharmacy, Amrita Vishwa Vidyapeetham, AIMS Health Sciences Campus, Kochi 682041, India; 2 Department of Pharmacy, and Research Institute of Life Pharmaceutical Sciences, Sunchon National University, Suncheon 57922, Korea; ddazzo005@naver.com; 3 Department of Pharmaceutics and Industrial Pharmacy, College of Pharmacy, Taif University, P.O. Box 11099, Taif 21944, Saudi Arabia; a.khamies@tu.edu.sa; 4 Department of Pharmaceutical Chemistry, College of Pharmacy, Jouf University, Sakaka 72341, Saudi Arabia; mhmdgwd@ju.edu.sa; 5 Department of Chemistry, Sri Venketeswara College, University of Delhi, New Delhi 110021, India; rangarajan93150@gmail.com; 6 Department of Pharmacognosy, Amrita School of Pharmacy, Amrita Vishwa Vidyapeetham, AIMS Health Sciences Campus, Kochi 682041, India; lekshmirnath@aims.amrita.edu; 7 Molecular Bio-Computation and Drug Design Laboratory, School of Health Sciences, Westville Campus, University of KwaZulu-Natal, Durban 4001, South Africa; clegoni@gmail.com (C.A.); soliman@ukzn.ac.za (M.E.S.S.); 8 Department of Pharmacology, Grace College of Pharmacy, Palakkad 678004, India; githaliz@gmail.com

**Keywords:** chalcone, MAO-B, selectivity, reversibility, cytotoxicity, ROS, molecular dynamics

## Abstract

To develop new potent and highly selective MAO-B inhibitors from chalcone-thioethers, eleven chalcones-thioethers were synthesized and their monoamine oxidase (MAO) inhibition, kinetics, reversibility, and cytotoxicity of lead compounds were analyzed. Molecular dynamics were carried out to investigate the interactions. Compound **TM8** showed potent inhibitory activity against MAO-B, with an IC_50_ value of 0.010 µM, followed by **TM1**, **TM2**, **TM7**, and **TM10** (IC_50_ = 0.017, 0.021, 0.023, and 0.026 µM, respectively). Interestingly, **TM8** had an extremely high selectivity index (SI; 4860) for MAO-B. Reversibility and kinetic experiments showed that **TM8** and **TM1** were reversible and competitive inhibitors of MAO-B with K_i_ values of 0.0031 ± 0.0013 and 0.011± 0.001 µM, respectively. Both **TM1** and **TM8** were non-toxic to Vero cells with IC_50_ values of 241.8 and 116.3 µg/mL (i.e., 947.7 and 402.4 µM), respectively, and at these IC_50_ values, both significantly reduced reactive oxygen species (ROS) levels. **TM1** and **TM8** showed high blood-brain barrier permeabilities in the parallel artificial membrane permeability assay. Molecular dynamics studies were conducted to investigate interactions between **TM1** and **TM8** and the active site of MAO-B. Conclusively, **TM8** and **TM1** are potent and highly selective MAO-B inhibitors with little toxicity and good ROS scavenging abilities and it is suggested that both are attractive prospective candidates for the treatment of neurological disorders.

## 1. Introduction

Parkinson’s disease (PD) is considered the second most common progressive neurodegenerative disorder in the elderly and is characterized by motor system disruption [1]. Over ten million people worldwide suffer from PD, which places an onerous responsibility on the medical community to develop an effective therapy [2]. This motor system disruption is caused by the extensive degeneration of the dopaminergic neurons, which leads dopamine (DA) depletion [3]. Current treatment strategies center on enhancing dopamine-like activities using monoamine oxidase (MAO)-B inhibitors, dopamine agonists, and catechol-O-methyltransferase (COMT) inhibitors, whereas developmental efforts appear to be focused on the identification of non-dopaminergic candidates like glutamate antagonists and anticholinergic drugs [4].

MAO is found in two isoforms, that is, MAO-A and MAO-B, and contains flavin adenine dinucleotide (FAD) as a cofactor. These isoforms share 70% amino acid sequence similarity and differ functionally in terms of their substrate specificities and inhibitor affinities [5]. Selective MAO-A and MAO-B inhibitors are considered important for the treatment of various neuropsychiatric and neurodegenerative disorders, respectively [6]. For example, the level of endogenous dopamine enhances the blockade of dopamine metabolism by selective MAO-B inhibition [7]. Numerous studies have documented that the formation of the 1-methyl-4-phenylpyridinium ion (MPP^+^; a bioactive metabolite of 1-methyl-4-phenyl-1,2,3,6-tetrahydropyridine (MPTP)) is catalyzed by MAO-B, and that MPP^+^ induces the neurotoxic effects associated with the pathophysiology of PD [8]. MPP^+^ interacts with mitochondrial complex 1 to induce neural toxicity in PD [9]. In view of the important roles played by MAO-B in neurodegenerative disorders, novel highly selective MAO-B inhibitors are of considerable interest [10].

A recent strategy used to produce highly selective MAO-B inhibitors included the installation of two aryl or heteroaryl rings on either end of the two to four carbons or nitrogen-containing spacer units, which include α-β-unsaturated ketones, pyrazolines, enamides, and hydrazones [11,12,13,14,15]. The presence of a π-conjugated system with rotatable bonds in the linker can provide different orientations and facilitate interactions between designed scaffolds and the active site of MAO-B [16]. Chalcones are considered versatile scaffolds for selective MAO-B inhibitors. Recently, Guglielmi et al. predicted that chalcone derivatives are likely to exhibit selective human MAO-B (hMAO-B) inhibition based on results obtained using the free online tool SwissTargetPrediction (www.swisstargetprediction.ch (accessed on 10 October 2021)), which supports our previous experimental results [17,18].

**Scheme 1:**
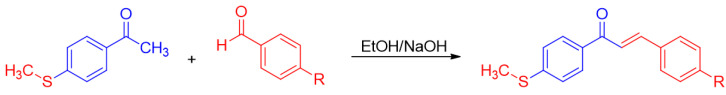
Synthetic route to chalcone-thiomethyl ethers **TM1-11**.

Chalcones are α-β unsaturated ketones with three rotatable bonds and are endowed with a number of central nervous system (CNS) related activities, which include adenosine receptor antagonist and neuroprotective activities, ability to cross the blood-brain barrier (BBB), and inhibitions of MAO-B, acetylcholine esterase (AChE), transglutaminase, and β-amyloid plaques formation [19,20,21,22]. Numerous studies have demonstrated that substituent selection and the orientations of substituents on the chalcone A and/or B rings provide a promising means of producing reversible and selective inhibitors of hMAO-A and -B. Notably, the presence of lipophilic electron-donating groups (i.e., ethyl, methoxy, methyl, and dimethylamino, or halogens) at the same position on the **B** ring results in hMAO-B inhibition in the nanomolar range [23,24,25,26], whereas the introduction of methoxy at *para*-position of the **A** ring is associated with hMAO-B inhibitory activity [27,28,29,30]. The design strategy used in the present study involved the isosteric replacement of the **A** ring methoxy with a thiomethyl group and the introduction of different substituents on the **B** ring (Scheme 1).

**Figure 1:**
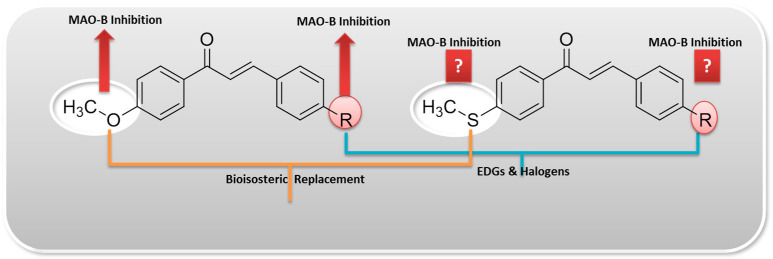
Design of chalcone-thioethers by bio-isosteric replacement for MAO-B inhibition.

To the best of our knowledge, the influence of isosteric replacement of the A-ring methoxy with a thiomethoxy group has not been previously reported to enhance hMAO-B inhibition (Figure 1).

We report a series of chalcone-thioether derivatives, which were prepared and evaluated for their MAO-A and MAO-B inhibitory activities, cytotoxicities, and effect on reactive oxygen species (ROS). Lead molecules were subjected to detailed molecular dynamics (MD) study to access their molecular mechanisms.

## 2. Results

### 2.1. MAO Inhibition

All eleven chalcone derivatives potently inhibited MAO-B at 10 µM; residual activities were <15% (Table 1). Compound **TM8** showed the highest inhibitory activity against MAO-B with an IC_50_ value of 0.010 µM, and **TM1**, **TM2**, **TM7**, and **TM10** also exhibited potent inhibitory effects (IC_50_ = 0.017, 0.021, 0.023 and 0.026 µM, respectively). The other six compounds had IC_50_ values < 0.15 µM. Interestingly, **TM1**, **TM2**, **TM4**, **TM7**, **TM8**, and **TM10** were 3.7, 3.0, 1.1, 2.7, 6.3 and 2.4 times, respectively, more potent than the reference drug lazabemide. Similarly, **TM1**, **TM2**, and **TM8** were 1.6, 1.3 and 2.8 times, respectively, more potent than pargyline. Compounds **TM7** and **TM10** were almost as potent as pargyline. Regarding the effects of substituents, the inhibitory potencies of these compounds followed the order –Cl > –H > –OH > –NO_2_> –F.

Compounds **TM5**, **TM6**, **TM2**, and **TM3** at 10 µM inhibited MAO-A with IC_50_ values of 5.91, 6.71, 7.45, and 8.82 µM, respectively, whereas the other seven compounds had lesser effects (residual activities > 50%) (Table 1). Interestingly, **TM8** had a much higher selectivity index (SI; 4,860) for MAO-B than the other compounds. The next highest were compounds **TM7** and **TM1** with SI values of 1304 and 812, respectively.

### 2.2. Kinetics

**Figure 2:**
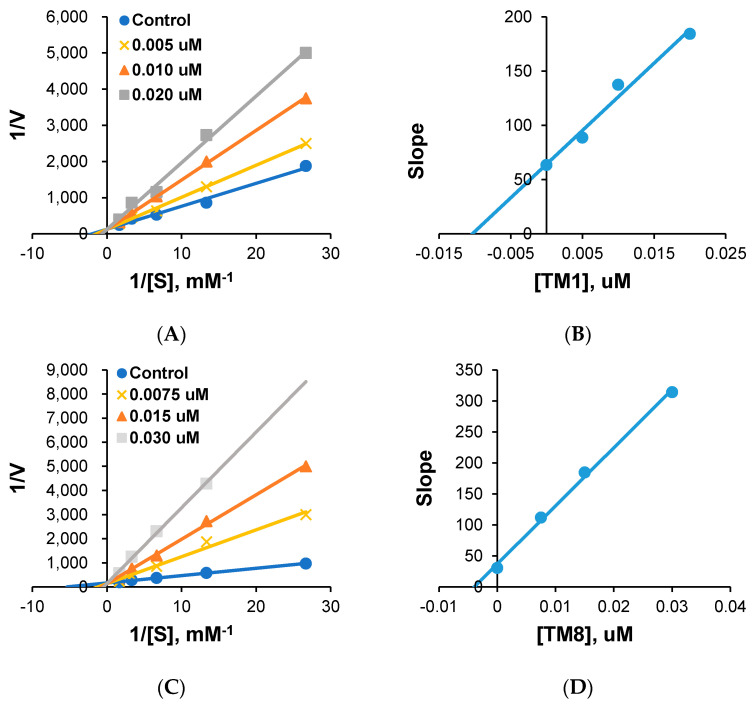
Lineweaver-Burk plots for MAO-B inhibition by **TM1** and **TM8** (**A**,**C**), and their respective secondary plots (**B**,**D**) of slopes vs. inhibitor concentrations.

Kinetic studies on MAO-B inhibitions were performed on **TM1** and **TM8**. Lineweaver-Burk plots and secondary plots showed that the lines intersected y-axes, thus indicating, **TM1** and **TM8** competitively inhibited MAO-B (Figure 2A,C). K_i_ values were 0.011 ± 0.001 and 0.0031 ± 0.0013 µM, respectively (Figure 2B,D). These results suggest that compounds **TM1** and **TM8** compete with the substrate at the active site of MAO-B.

### 2.3. Reversibility

**Figure 3:**
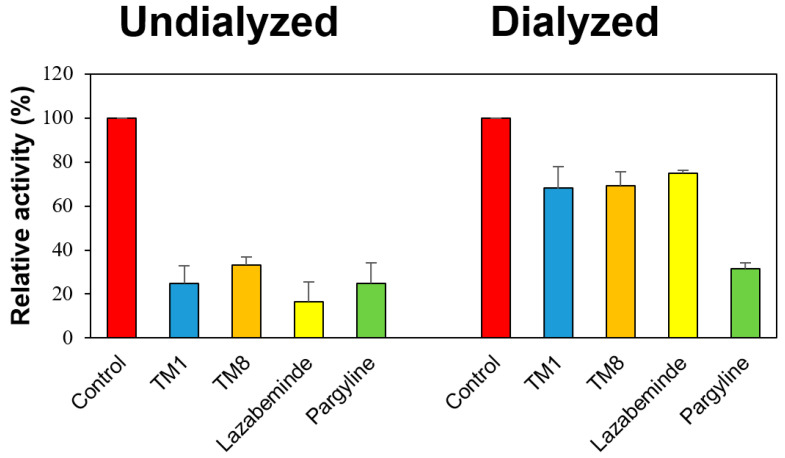
Dialysis recoveries of MAO-B inhibitions by **TM1** and **TM8.**

MAO-B inhibition reversibility studies were conducted on compounds **TM1** and **TM8** using a dialysis-based method. MAO-B inhibition by **TM1** was recovered by dialysis from 24.9% (A_U_) to 68.1% (A_D_), and inhibition by **TM8** was recovered from 33.1% to 69.3%. These recoveries were similar to that observed for the reference reversible lazabemide (from 16.6% to 74.9%). Inhibition of MAO-B by pargyline (the reference irreversible inhibitor) was not recovered (from 24.7% to 31.4%) (Figure 3). These results showed that compounds **TM1** and **TM8** were reversible MAO-B inhibitors.

### 2.4. Cytotoxicity Evaluation

**Figure 4:**
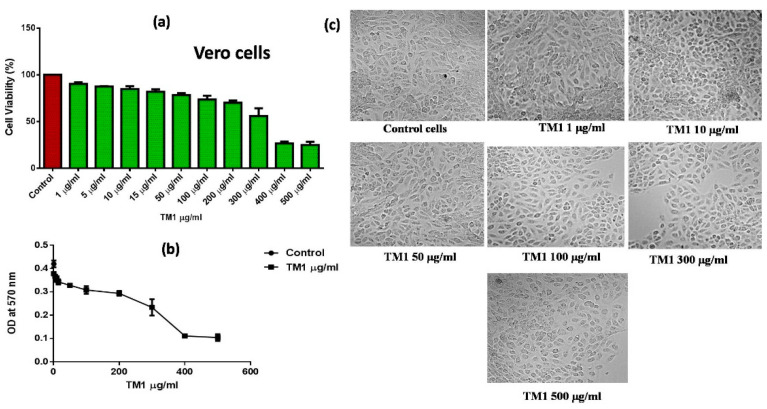
Effect of **TM1** on Vero cell viability. (**a**) cell viability treated with **TM1**; (**b**) dose-response curve; (**c**) phase contrast microscope images of Vero cells treated with different concentrations of **TM1**. Cells were treated with **TM1** for 48 h and cell viabilities were assessed by MTT assay as described in Section 4.

**Figure 5:**
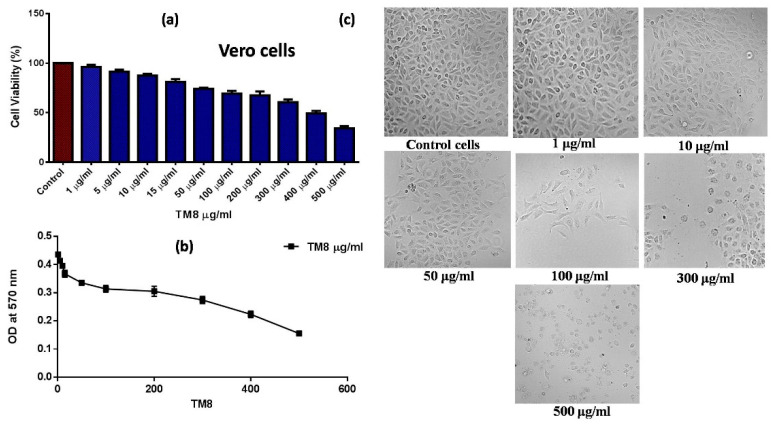
Effect of **TM8** on Vero cell viability. (**a**) cell viability treated with **TM8**; (**b**) dose-response curve; (**c**) phase contrast microscope images of Vero cells treated with different concentrations of **TM8**. Cells were treated with **TM8** for 48 h and cell viabilities were assessed by MTT assay as described in Section 4.

African Green Monkey kidney cells (VERO cells) were used to evaluate the cytotoxic effects of **TM1** and **TM8.** Cells were incubated with different doses (1 µg/mL to 500 µg/mL) of the two compounds for 48 h, and relative cell viabilities were determined by MTT assay. Compound **TM1** and **TM8** reduced viabilities concentration-dependently (Figure 4a and Figure 5a); **TM1** and **TM8** treatments resulted in high viability (>90%) at concentrations <250 and <100 µg/mL (i.e., 983.1 and 346.3 µM), respectively, and the IC_50_ values were 241.6 and 116.2 µg/mL (i.e., 947.7 and 402.4 µM), respectively (Figure 4b and Figure 5b). Phase contrast microscopy showed that treatments above IC_50_ values resulted in membrane damage and reduced cell numbers. These results suggest that **TM1** and **TM8** are safe at low concentrations (Figure 4c and Figure 5c), and that at concentrations > their IC_50_ values induced oxidative stress.

### 2.5. ROS Assay

**Figure 6:**
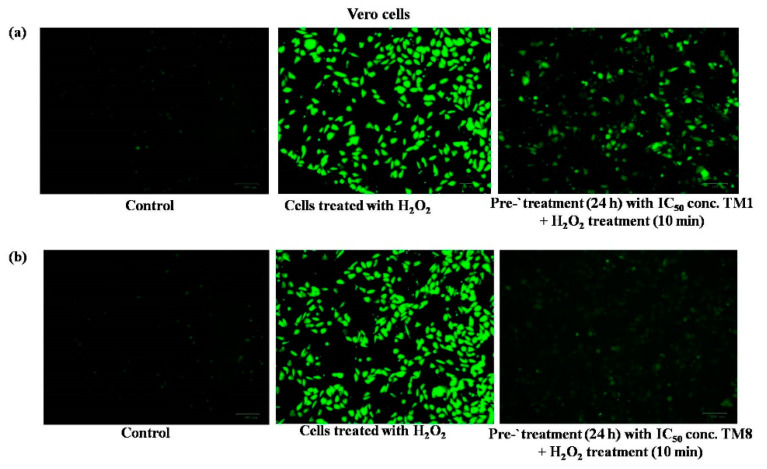
Effects of **TM1** (**a**) and **TM8** (**b**) on H_2_O_2_-induced ROS levels. Vero cells (1 × 10^6^ cells/mL) were pre-incubated with 100 μg/mL of 30% H_2_O_2_ and ROS production was evaluated immediately as described in Section 4.

ROS increase vulnerability to neuronal damage by causing oxidative damage in the brain during neurodegenerative diseases. When Vero cells were exposed to exogenous H_2_O_2_, intracellular ROS levels were elevated, but treatments with **TM1** or **TM8** at their IC_50_ values resulted in appreciably lower ROS levels (Figure 6a,b).

### 2.6. Blood-Brain Barrier (BBB) Permeation Study by Parallel Artificial Membrane Permeability Assay (PAMPA)

A highly effective permeability and high CNS bioavailability of thiomethane-containing chalcones were demonstrated with Pe ranges between 16.26 and 13.28 × 10^−6^ cm/s (Table 2) in the parallel artificial membrane permeability assay (PAMPA).

### 2.7. Inhibitor-Induced Binding Pocket Dynamics

**Figure 7:**
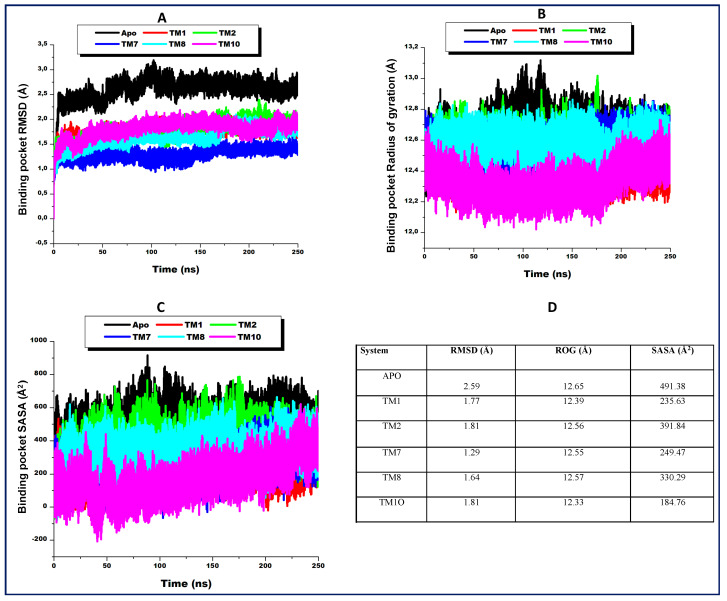
Comparative RMSD plots (**A**), comparative radii of gyration plots (**B**), and comparative SASA plots (**C**) of the binding site residues of free MAO-B (black) and MAO-B bound to TM1 (red), TM2 (green), TM7 (blue), TM8 (cyan), or TM10 (magenta), and average RMSD, ROG, and SASA values of MAO-B binding site residues (**D**).

**Figure 8:**
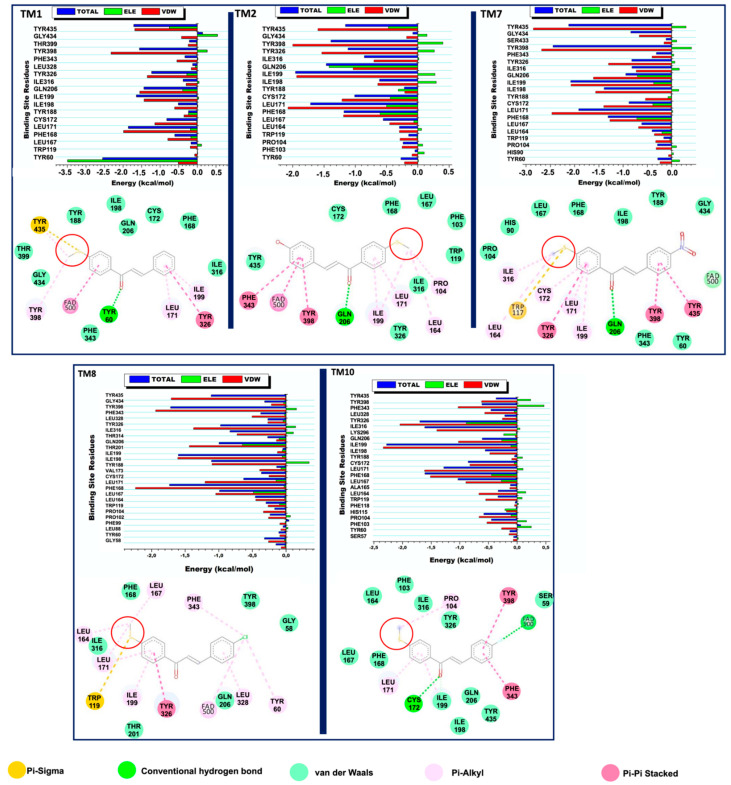
Per-residue energy contributions of interacting residues at the MAO-B inhibitor binding site after **TM1**, **TM2**, **TM7**, **TM8** or **TM10** binding. Corresponding residue interactions are also shown. Inserts highlight the interaction dynamics of the SCH3 moeity of each compound.

Binding pocket residues of MAO-B exhibited average root mean square deviations (RMSDs), of 1.77, 1.81, 1.29, 1.64 and 1.81 Å for **TM1**, **TM2**, **TM7**, **TM8** and **TM10**, respectively (Figure 7), suggesting relatively stable conformations since average values were below 2 Å. An average RMSD of 2.59 Å was estimated for unbound MAO-B, which suggested a relatively unstable conformation. According to Luque and Freire in 2000 [19], a stable binding pocket conformation provides optimum binding affinity for small ligands. We assessed the rigidity or compactness of the binding pocket residues by estimating their conformational changes within the binding pocket of MAO-B mediated by **TM1, TM2, TM7, TM8** and **TM10**, which altered the overall structure and function of MAO-B radii of gyration over a 250 ns MD simulation period. Relatively stable conformations were observed, and binding pocket residues in inhibitor-bound MAO-B complexes were more compact/rigid with average radii of gyration (ROGs) of 12.39, 12.56, 12.55, 12.57 and 12.33 Å for **TM1**, **TM2**, **TM7**, **TM8** and **TM10**, respectively. Unbound MAO-B binding pocket residues exhibited a relatively higher average ROG of 12.65 Å, which suggest that bindings of **TM1**, **TM2**, **TM7**, **TM8** and **TM10** enhanced binding pocket stability. An assessment of the solvent surface exposure of binding pocking residues also provided insights into the conformational dynamics of the binding pocket after inhibitor binding [14,20,21]. As shown in Figure 8, binding pocket residues in inhibitor-MAO-B complexes exhibited lower average solvent accessible surface area (SASA) values of 235.63, 391.84, 249.47, 330.29 and 184.76 Å^2^ for **TM1**, **TM2**, **TM7**, **TM8** and **TM10**, respectively, while the unbound conformation had an SASA of 491.38 Å^2^. This observation was in line with ROG results and indicated MAO-B/TM series binding resulted in greater structural compactness and inward folding of the binding pocket, which also suggests enhanced ligand binding and stability.

## 3. Discussion

Chalcones provide a simple framework with broad-spectrum biological activities and are accessible by Claisen-Schmidt condensation. The aldehyde used becomes the **B** ring of the chalcone, whereas the acetophenone used becomes the **A** ring. The α,β-unsaturated ketone moiety of chalcones is considered a Michael acceptor and may be involved in the signaling of many biochemical pathways. The presence of olefinic linkage introduces the possibility of *cis*- and *trans*-isomers, but the more thermodynamically stable *trans*-form is invariably produced. The presence of electron-donating and -withdrawing groups on the phenyl ring of chalcones greatly influences the electron density on the enone moiety and the orientations of the three rotatable bonds of the chalcone framework. These unique features enable this simple framework to interact with enzyme targets, preferably those with hydrophobic pockets.

**Figure 9:**
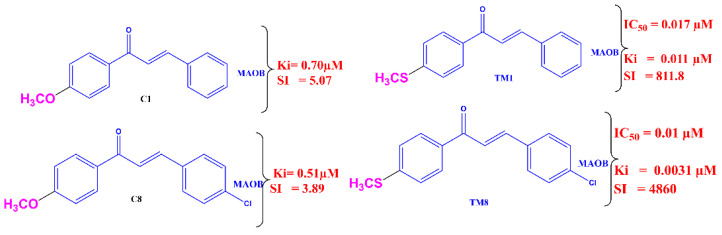
Influence of bio-isosteric replacement with respect to MAO-B inhibition.

Our structure-activity relationship (SAR) investigation of the designed chalcone-thioethers showed reasonable relations with observed inhibitory effects. In the 11 chalcone derivatives, the thioether (-SMe) group was located on the **A** ring and other alterations were made by placing various substituents at the *para*-position on the ring **B**. Compound **TM1** (the parent compound) had no substituent at the *para*-position but had excellent MAO-B inhibitory effect with an IC_50_ value of 0.017 ± 0.0026 µM and an SI of 811.8. The K_i_ value of **TM1** for MAO-B (0.011 ± 0.001 µM) is much potent than the unsubstituted methoxylated chalcone (**C1**) previously reported by our group (0.70 ± 0.05 µM) [30]. This crucial observation clearly showed that bio-isosteric replacement with thioether group on the *para* position of phenyl ring **A** in the chalcone framework resulted in pronounced MAO-B inhibitory activities. The introduction of chlorine atom on the *para* position of ring **B** with thioether group on the **A** ring resulted highly potent MAO-B inhibition (K_i_ = 0.0031µM) (Figure 9).

**Figure 10:**
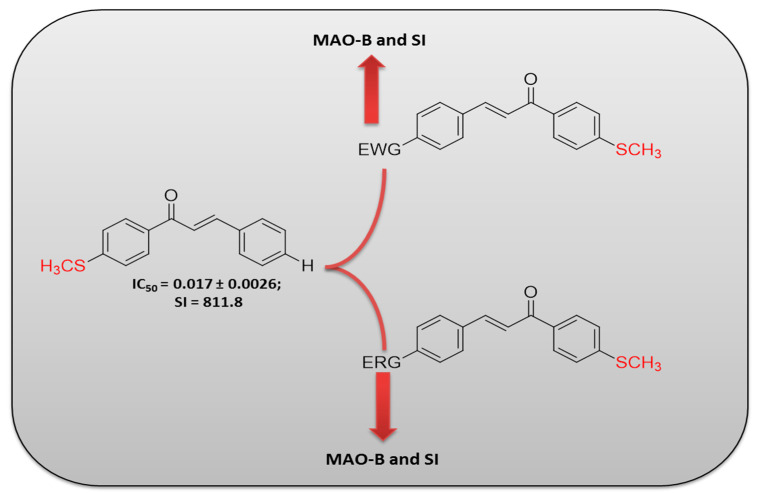
SARs of chalcone-thioethers with respect to MAO-B inhibition.

When –NMe_2_ (a strong election-releasing group (ERG)) was introduced, resulting **TM5** showed a 7-fold fall in inhibitory activity and a 10-fold reduction in selectivity (0.12 ± 0.01 µM and 49.3, respectively). Similar results were observed when -CH_2_CH_3_ (a weak ERG) was introduced to produce **TM6** (Table 1), which was ascribed to its bulkiness. The results were ascertained by introduction of small but strong and relatively week electron-releasing groups such as –OH, –OMe, and –Me. The –OMe (a strong ERG) increased inhibition and selectivity with respect to **TM5** and **TM6** (IC_50_ = 0.088 ± 0.011 µM and 100.2). Of the ERG introduced, those with a weak ERG (e.g., –Me), except -OH, increased inhibition and selectivity (IC_50_ = 0.058 ± 0.001 µM and 206.9, respectively). The strong ERG –OH, **TM2**, had an inhibitory effect (IC_50_ = 0.021 ± 0.003 µM) similar to the parent compound **TM1**, but considerably less selectivity, which may have been due to hydrogen bonding. Generally, ERGs had marked MAO-B inhibitory effects as compared with the parent compound **TM1**. The effects of electron-withdrawing groups (EWG) on chalcone-thioethers on MAO-B inhibitory activity were also investigated. The introduction of –NO_2_ (the strongest EWG) in **TM7** resulted in inhibitory activity (IC_50_ = 0.023 ± 0.001 µM) similar to the parent compound **TM1** but improved selectivity (1,304.3), whereas the introduction of -CF_3_ (a weak EWG) in **TM11** caused a considerable drop in inhibitory activity and selectivity (IC_50_ = 0.081 ± 0.032 µM and 311.1, respectively). Other halogens (also EWGs) such as –F, –Cl, and –Br had effects similar to **TM10** (IC_50_ = 0.026 ± 0.004 µM) and a drop (0.088 ± 0.006 µM) in MAO-B inhibition effect with respect to the parent compound, except –Cl (**TM8**). In addition, a considerable drop in selectivity was also observed for these groups. The –Cl was a more effective pharmacophore than other halogens and EWGs. **TM8** containing-Cl had the highest MAO-B inhibitory activity (IC_50_ = 0.010 ± 0.005 µM) and selectivity (4860.0) and had 6.3 and 2.8 times more inhibitory activity than the reference drugs lazabemide and pargyline, respectively. In contrast, strong EWGs such as –OH, –OMe, –NMe_2_, and –Et exhibited better MAO-A inhibitory effects than the parent compound and other EWGs but had far lower inhibitory effects than the reference drugs (Table 1). SAR in this study can be summarized as shown in Figure 10.

Previously, our group developed some methoxylated chalcones as selective MAO-B inhibitors and the lead molecule showed effective inhibitory activity (IC_50_ = 0.29 ± 0.01 µM) [30]. In current study, the bioisosteric replacement of methoxy group into thiomethyl group showed significantly increased MAO-B inhibition. In another recent study of Shalaby et al., compound (E)-3-(3-bromophenyl)-1-(4-(methylthio)phenyl)prop-2-en-1-one exhibited efficient MAO-B inhibition with IC_50_ = 0.19 ± 0.01 µM [25]. It is noteworthy that the replacement of methoxy chalcones to thiomethyl resulted in enhancement of overall MAO-B inhibitory profiles with high SI values. Interestingly, the analogues tested resulted in potent inhibitory activities in nanomolar ranges towards MAO-B inhibition.

Toxicity assessment were conducted using Vero cells, which are commonly used to assess viability and lethality at a cellular level. We found that more than 90% of cells treated with **TM1** or **TM8** at effective concentrations remained viable. However, extensive blebbing and membrane damage were observed when cells were treated at high concentrations. Our cell viability and phase contrast microscopy studies showed that **TM1** is a safer proposition than **TM8**. Furthermore, our investigation of the inhibitory effects of **TM1** and **TM8** on ROS, which are known to play significant roles in the pathogeneses of neurodegenerative diseases and to cause cell death or oxidative stress (OS) when present in excess, showed **TM1** and **TM8** at their IC_50_ levels mitigated H_2_O_2_-induced increases in ROS levels.

MD simulation provides a means of exploring the structural changes and functions caused by the binding of small molecules and has been used to investigate associations between protein activities and structural dynamics. By comparing protein conformations in binding pockets before and after ligand binding, we were able to identify inhibitor-induced conformational changes associated with inhibition of MAO-B. To observe these conformational changes, we employed MD simulation metrics, that is, RMSD, ROG, and SASA.

Having established that compound bindings induced conformational changes that might favor binding affinity, we estimated the binding free energy for compound/MAO-B complexes using experimentally determined IC_50_ values. Using the MM/PBSA approach, we calculated the binding energies of the compounds (Table 1). As shown by the table, the compounds exhibited favorable total binding energies, which was in line with our experimental data. Total binding energies for **TM1**, **TM2**, **TM7**, **TM8**, and **TM10** were −40.83 ± 2.63, −34.40 ± 2.63, −47.02 ± 2.65, −37.02 ± 3.05, and −35.14 ± 2.81 kcal/mol, respectively, which suggested that these chalcone derivatives engage in favorable interactions with the active site residues of MAO-B. Though all the compounds exhibited binding in accord with their free total binding energies, **TM7**/MAO-B complex had the highest free total binding energy *(*−47.02 ± 2.65 kcal/mol), which was attributed to greater Van der Waals (VDW) forces and electrostatic (ELE) interactions. Overall, it was noticed that VDW interactions (∆EvdW) were important for ligand binding, whereas the favorable ELE interactions (∆Eele) compensated for unfavorable polar solvation energies.

Intermolecular interactions between compounds and binding site residues influence the stabilities of the complexes formed. The total binding free energies of the compounds **TM**1 and **TM**8 were decomposed into individual residue-based contributions using the MM/PBSA method. The VDW and ELE energy contributions were expressed as percentages of total binding free energies to determine which residues and energy constituents had the greatest effects on total energy. As shown in Figure 8, the top three residues that contributed the most to the binding of **TM1** against MAO-B were TYR60 (−2.55 kcal/mol), LEU171 (−1.85 kcal/mol), and TYR435 (−1.71 kcal/mol); that to the binding of **TM2** were ILE199 (−1.95 kcal/mol), LEU171 (−1.71 kcal/mol), and GLN206 (−1.46 kcal/mol); that to the binding of **TM7** were TYR398 (−2.44 kcal/mol), TYR435 (−2.12 kcal/mol), and ILE199 (−2.0 kcal/mol); that to the binding of **TM8** were LEU171 (−1.73 kcal/mol), TYR398 (−1.71 kcal/mol) and ILE199 (−1.60 kcal/mol); and that to the binding of **TM10** were ILE199 (−2.27 kcal/mol), TYR326 (−1.69 kcal/mol), and ILE316 (−1.61 kcal/mol). Although unfavorable ELE interactions were observed in interaction profiles, as shown by the plots in Figure 8, these would have had little effects on total binding due to the cumulative effects of ELE and VDW energy contributions. Particular focus on the SCH3 group, as highlighted in Figure 8, showed the formation of high-affinity interactions with MAO-B, relative to other moieties thus indicating its cruciality to the binding mechanism of synthesized compounds. Specific interactions of the SCH3 group in each compound included; TM1 (π-sigma and π-alkyl with TYR435, π-alkyl with TYR398), TM2 (π-alkyl with ILE199, LEU164 and PRO104), TM7 (π-sigma with TRP117 and π-alkyl with TRP117 and TRP316), TM8 (π-sigma with TRP119 and π-alkyl with LEU164, LEU171, LEU167) and TM10 (π-alkyl with PRO104). These SCH3-mediated high-affinity interactions could contribute to stability and the overall total binding affinity of each compound.

Study of the residues involved in binding revealed that the comparatively high binding affinity of **TM7** could be attributed to relatively high binding energy contributions. Furthermore, these cumulative per-residue energy contributions might explain the MAO-B inhibitory effects of **TM1**, **TM2**, **TM7**, **TM8**, and **TM10**. Also, these estimations support predictions based on binding free energies.

## 4. Materials and Methods

### 4.1. Synthesis

Chalcone-thioethers (**TM1-11**) were synthesized using base-catalyzed Claisen-Schmidt condensation between various *para*-substituted aromatic aldehydes and 4-thiomethyl acetophenone. Target compounds were produced by stirring ethanol solutions of 0.01 M of 4-thiomethylacetophenone with appropriate aromatic aldehydes containing 40% NaOH solution for 8–10 h [32]. The resulting reaction mixtures were poured into ice-cold water, and the products were collected, washed thoroughly with water, and recrystallized from ethanol. Spectral data were provided in Supplementary Materials.

### 4.2. Biological Evaluations

#### 4.2.1. MAO Enzyme Inhibition

MAO-A activity was estimated continuously for 20 min at 316 nm using 0.06 mM kynuramine as substrate, whereas MAO-B activity was measured for 30 min at 250 nm using 0.3 mM benzylamine as substrate, as described previously [33]. MAO activity assays were performed using recombinant human MAO-A or MAO-B.

#### 4.2.2. Kinetics Study

MAO-A activities were estimated after exposing it to inhibitors at 10.0 μM, whereas MAO-B activities were estimated at an inhibitor concentration of 1.0 μM. IC_50_ values were determined by measuring residual enzyme activities. Toloxatone and lazabemide were used as reference reversible inhibitors for MAO-A and MAO-B, respectively, and clorgyline and pargyline as reference irreversible inhibitors. K_i_ values and inhibitor types were determined by kinetic testing, as previously described [34]. Kinetic tests were performed at 5 different substrate concentrations and inhibitor concentrations of 0, ~1/2 × IC_50_, IC_50_, and 2 × IC_50_ values. Lineweaver-Burk plots (LB) and their secondary plots were used to determine K_i_ values and inhibitor types.

#### 4.2.3. Reversibility Studies

The reversibilities of MAO-B inhibitions by **TM1** and **TM8** were assessed by dialysis. Test compounds were preincubated with MAO-B at 0.15 µM for 30 min, as described previously [35]. MAO-B was also preincubated with the positive controls for the experiment; lazabemide (a reference reversible MAO-B inhibitor) or pargyline (a reference irreversible MAO-B inhibitor) at 0.20 and 0.30 µM, respectively. Reversibility patterns were determined by comparing the activities of dialyzed (A_D_) and undialyzed (A_U_) samples.

#### 4.2.4. Cytotoxicity and ROS Assays

The cytotoxicities and ROS quenching abilities of the two lead compounds (**TM1** and **TM8**) were evaluated as previously described [36,37].

#### 4.2.5. BBB Study by PAMPA Method

The CNS bioavailability of the lead candidates was further ascertained by PAMPA method [31].

### 4.3. Computational Studies

#### 4.3.1. System Preparation

Initial coordinates of MAO-B (PDB ID: 6RKB) were selected [38]. The structure of MAO-B consists of two identical chains, A and B, which forms a MAO-B homodimer. Each chain contains a cofactor FAD, an inhibitor, water, and non-standard residues. Chain B was removed together with the water and non-standard residues using UCSF Chimera [39], and the ‘cleaned’ chain A was then saved in pdb format. The 2D structures of the compounds **TM1**, **TM2**, **TM7**, **TM8** and **TM10** were drawn using Marvin Sketch and optimized with Avogadro to generate 3D structures. Using UCSF Chimera, hydrogen atoms and Gasteiger charges were added and results were saved in mol2 format for molecular docking at the active site of ‘cleaned’ MAO-B.

#### 4.3.2. Molecular Docking

Docking was performed using the AutoDock Vina module incorporated into UCSF Chimera [40]. A grid box with coordinates of 51.3568, 154.894 and 29.7124, and x, y, and z dimensions of 23.009, 22.8623, and 26.0373 was used to define the location of the MAO-B binding site. Docked results generated were saved in pdbqt format. Conformations with best binding scores were selected and saved.

#### 4.3.3. Molecular Dynamics Simulation

Six systems (five complexes bound with the inhibitors and unbound MAO-B (Apo)) were set up for molecular simulation using the GPU version incorporated in AMBER18 [41]. Forcefield FF14SB was used to define the systems. The ANTECHAMBER module was used to add atomic partial charges to ligands using the Restrained Electrostatic Potential (RESP) and the General Amber Force Field (GAFF) protocols [42].

## 5. Conclusions

The current study demonstrated a series of thiomethyl group containing chalcones as potent and selective class of MAO-B inhibitors. In conclusion, **TM8** and **TM1** were found to be reversible, selective, and competitive inhibitors of MAO-B with K_i_ values of 0.0031 ± 0.0013 and 0.011 ± 0.001 µM, respectively. The isosteric replacement of the **A** ring methoxy- with a thiomethyl- group and the introduction of different substituents on the **B** ring showed good MAO-B inhibition. Moreover, **TM8** and **TM1** were found to be safe as analyzed by an in vitro toxicity study. Additionally, the prooxidant and antioxidant levels can be retained by **TM8** and **TM1.** Our findings show that **TM8** and **TM1** are selective inhibitors of MAO-B and have potential therapeutic value for the treatment of neurological disorders.

## Figures and Tables

**Table 1:** Inhibitions of recombinant human MAO enzymes by Chalcone-thioethers ^a^.

Compound	Residual Activity at 10 µM (%)		IC_50_ (µM)		SI ^b^
	MAO-A	MAO-B	MAO-A	MAO-B	
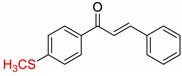 **TM1**	62.6 ± 9.7	13.2 ± 0.7	13.80 ± 0.05	0.017 ± 0.003	811.8
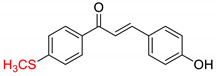 **TM2**	48.3 ± 2.4	−5.7 ± 5.3	7.45 ± 0.25	0.021 ± 0.003	354.8
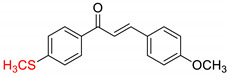 **TM3**	44.6 ± 1.9	6.9 ± 0.0	8.82 ± 0.09	0.088 ± 0.011	100.2
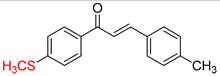 **TM4**	71.6 ± 3.8	14.7 ± 2.8	12.00 ± 0.99	0.058 ± 0.001	206.9
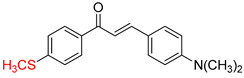 **TM5**	47.9 ± 3.7	1.6 ± 1.1	5.91 ± 0.87	0.120 ± 0.011	49.3
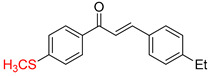 **TM6**	49.4 ± 8.7	−9.5 ± 0.0	6.71 ± 0.75	0.130 ± 0.005	51.6
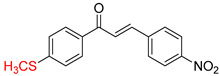 **TM7**	66.1 ± 2.6	6.0 ± 1.9	30.00 ± 3.02	0.023 ± 0.001	1304.3
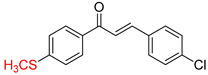 **TM8**	77.4 ± 8.2	4.1 ± 0.5	48.60 ± 3.44	0.010 ± 0.005	4860.0
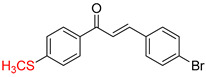 **TM9**	74.7 ± 0.0	7.5 ± 2.1	33.60 ± 2.56	0.088 ± 0.006	381.8
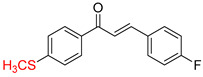 **TM10**	57.9 ± 6.0	−0.4 ± 2.7	13.70 ± 1.22	0.026 ± 0.004	526.9
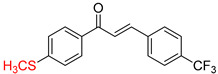 **TM11**	79.5 ± 0.7	7.4 ± 1.2	25.20 ± 0.14	0.081 ± 0.032	311.1
Toloxatone			1.08 ± 0.03	-	
Lazabemide			-	0.063 ± 0.015	
Clorgyline			0.0070 ± 0.0007	-	
Pargyline			-	0.028 ± 0.004	

^a^ Results are the means ± standard errors of duplicate experiments. ^b^ hMAO-B selectivity indices were calculated by dividing IC_50_(hMAO-A) by IC_50_(hMAO-B). Values for reference compounds were determined after preincubation with enzymes for 30 min.

**Table 2:** BBB assay of lead molecules of chalcone-thioethers by PAMPA method.

Compounds	Bibliography [31] Pe (×10^−6^ cm·s^−1^)	Experimental Pe (×10^−6^ cm·s^−1^)	Prediction
**TM1**		15.54 ± 0.33	CNS+
**TM2**		13.28 ± 0.80	CNS+
**TM7**		15.53 ± 0.71	CNS+
**TM8**		16.26 ± 0.26	CNS+
**TM10**		15.17 ± 0.44	CNS+
Progesterone	9.3	9.32 ± 0.33	CNS+
Verapamil	16.0	15.62 ± 0.42	CNS+
Piroxicam	2.5	2.44 ± 0.26	CNS±
Lomefloxacin	1.1	1.22 ± 0.02	CNS−
Dopamine	0.2	0.22 ± 0.01	CNS−

CNS+ (high BBB permeation predicted): Pe (10^−6^ cm·s^−1^) > 4.00; CNS− (low BBB permeation predicted): Pe (10^−6^ cm·s^−1^) < 2.00; CNS± (BBB permeation uncertain): Pe (10^−6^ cm·s^−1^) from 2.00 to 4.00.

## Data Availability

Data are contained within the article and Supplementary Materials.

## Supplementary Materials

The following are available online at https://www.mdpi.com/article/10.3390/ph14111148/s1, Figure S1: Compound TM1 1H-NMR spectrum, Figure S2: Compound TM1 ESI MS spectrum, Figure S3: Compound TM8 1H-NMR spectrum, Figure S4: Compound TM8 ESI MS spectrum, Figure S5: Compound TM9 1H-NMR spectrum, Figure S6: Compound TM9 ESI MS spectrum, Figure S7: Compound TM10 1H-NMR spectrum, Figure S8: Compound TM10 ESI MS spectrum.
