# Supplementary material for: Replacement of Chalcone-Ethers with Chalcone-Thioethers as Potent and Highly Selective Monoamine Oxidase-B Inhibitors and Their Protein-Ligand Interactions
Source: Pharmaceuticals (Basel). 2021 Nov 11;14(11):1148. doi: 10.3390/ph14111148 (PMC8623647; doi:10.3390/ph14111148)
Supplement: Supplementary file 1 [file pharmaceuticals-14-01148-s001.zip › pharmaceuticals-1446374-supplementary.pdf]

Supplementary Materials

Replacement of Chalcone-Ethers with Chalcone-Thioethers as Potent and Highly Selective Monoamine Oxidase-B Inhibitors and Their Protein-Ligand Interactions

Bijo Mathew [1,*,†], Jong Min Oh [2,†], Ahmed Khames [3], Mohamed A. Abdelgawad [4], T. M. Rangarajan [5], Lekshmi R. Nath [6], Clement Agoni [7], Mahmoud E. S. Soliman [7], Githa Elizabeth Mathew [8] and Hoon Kim [2,*]

1 Department of Pharmaceutical Chemistry, Amrita School of Pharmacy, Amrita Vishwa Vidyapeetham, AIMS Health Sciences Campus, Kochi-682 041, India
2 Department of Pharmacy, and Research Institute of Life Pharmaceutical Sciences, Sunchon National University, Suncheon 57922, Korea; ddazzo005@naver.com
3 Department of Pharmaceutics and Industrial Pharmacy, College of Pharmacy, Taif University, P.O. Box-11099, Taif-21944, Saudi Arabia; a.khamies@tu.edu.sa
4 Department of Pharmaceutical Chemistry, College of Pharmacy, Jouf University, Sakaka, Al Jouf 72341, Saudi Arabia; mhmdgwd@ju.edu.sa
5 Department of Chemistry, Sri Venketeswara College, University of Delhi, New Delhi-110021, India; rangarajan93150@gmail.com
6 Department of Pharmacognosy, Amrita School of Pharmacy, Amrita Vishwa Vidyapeetham, AIMS Health Sciences Campus, Kochi-682 041, India; lekshmirnath@aims.amrita.edu
7 Molecular Bio-computation and Drug Design Laboratory, School of Health Sciences, University of KwaZulu-Natal, Westville Campus, Durban 4001, South Africa; clegoni@gmail.com (C.A.); soliman@ukzn.ac.za (M.E.S.S.)
8 Department of Pharmacology, Grace College of Pharmacy, Palakkad 678004, India; githaliz@gmail.com (G.E.M.)
* Correspondence: bijomathew@aims.amrita.edu or bijovilaventgu@gmail.com (B.M.); hoon@sunchon.ac.kr (H.K.)
† These authors contributed equally to this work.

(2*E*)-1-[4-(methylsulfanyl)phenyl]-3-phenylprop-2-en-1-one (**TM1**):^{1}H-NMR (500 MHz, $CDCl_3$) δ: 2.53 (3H, s, S-CH_3), 7.32–7.30 (2H, d, H 2& H6), 7.43-7.41 (3H, m, H3, H4, H5), 7.53–7.50 (1H, d, J = 15.0 Hz, -CHα), 7.65–7.63 (2H, d, H3'&H5'), 7.82–7.79 (1H, d, J = 15.0 Hz, -CHβ), 7.97–7.95 (2H, d, H2'&H6'). ESI-MS (*m/z*): Calculated- 254.3467, Observed-254.3464.

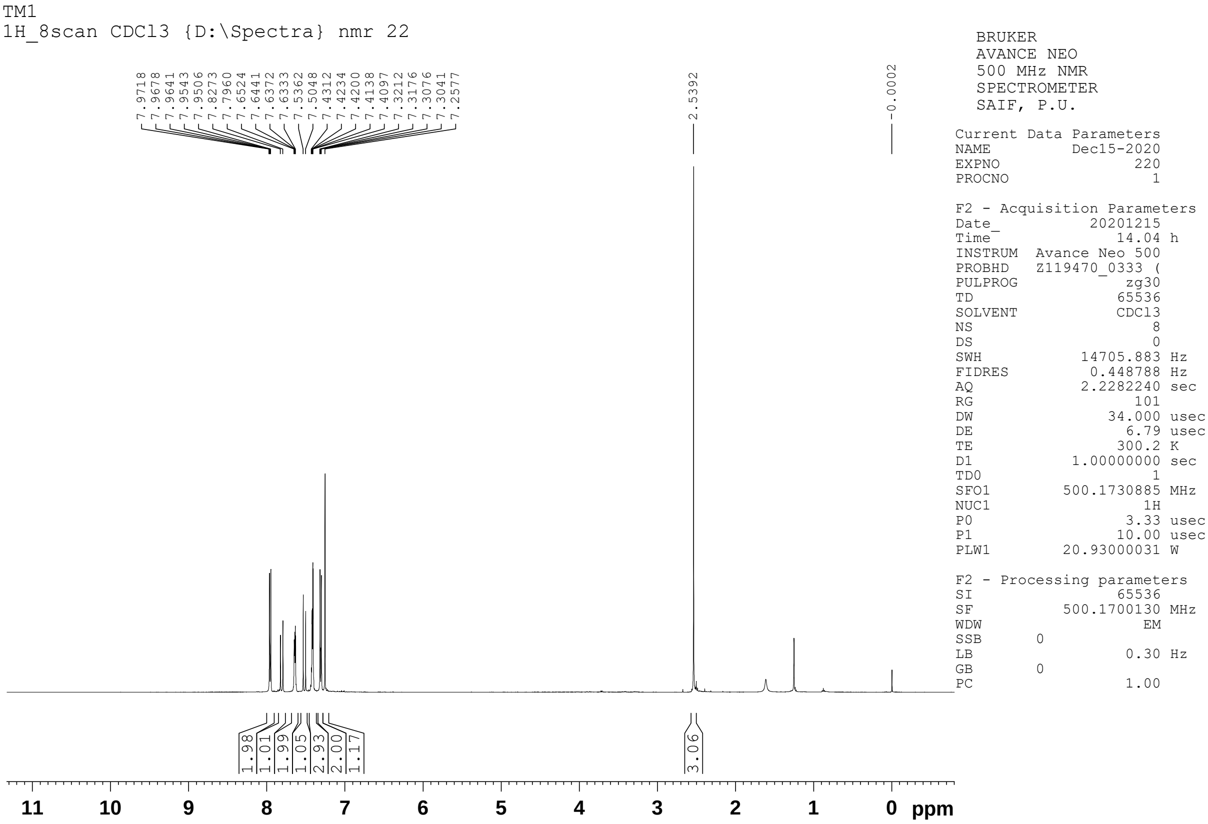

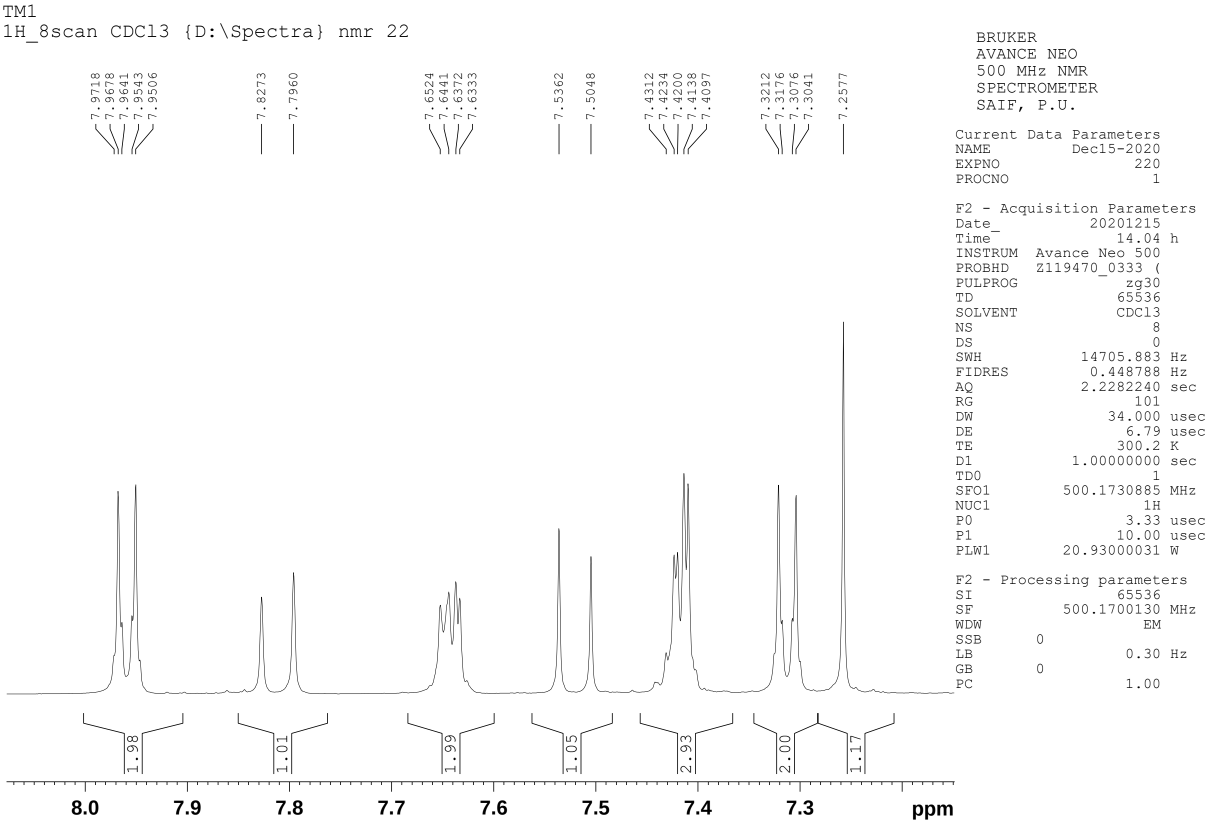

Figure S1. Compound **TM1** ^{1}H-NMR spectrum

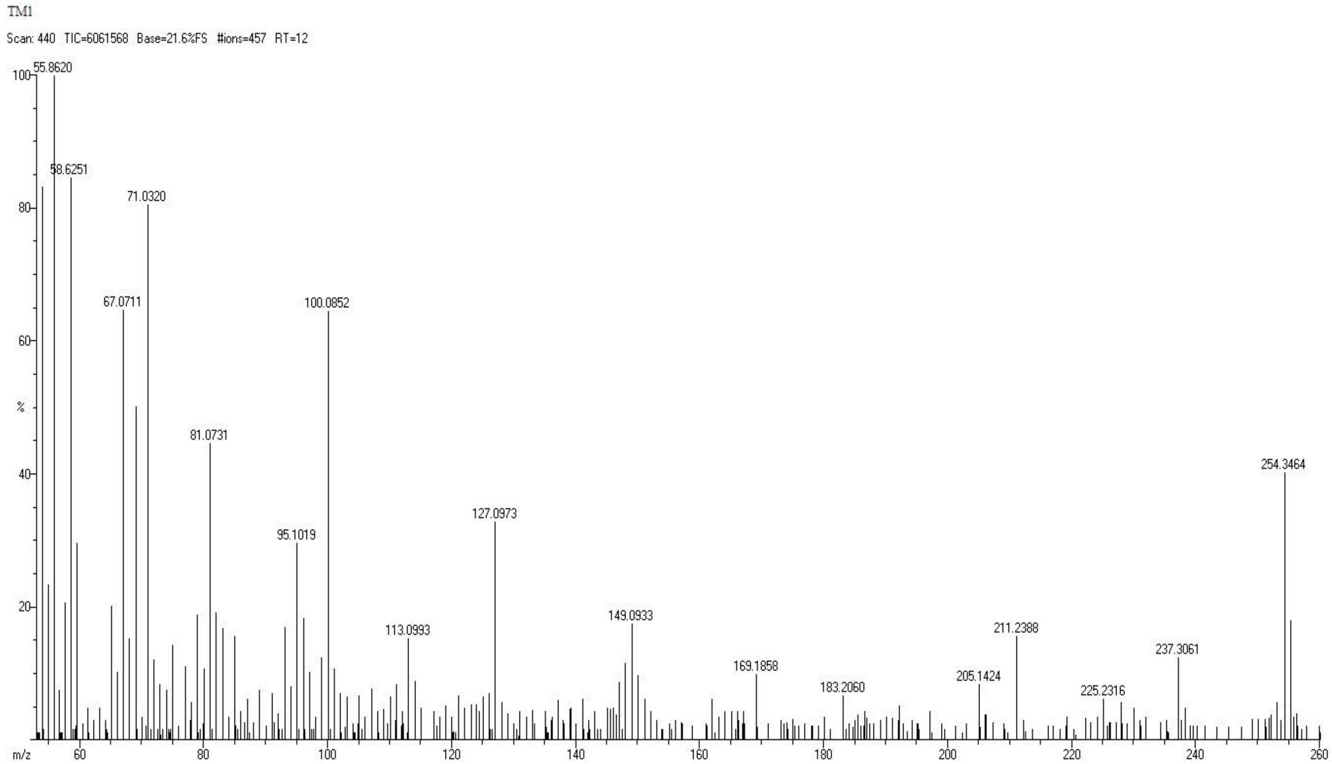

Figure S2. Compound **TM1** ESI MS spectrum

(2*E*)-3-(4-chlorophenyl)-1-[4-(methylsulfanyl)phenyl]prop-2-en-1-one **(TM8)**: ^{1}H-NMR (400 MHz, $CDCl_3$) δ: 2.54 (3H, s, S-CH_3), 7.32–7.30 (2H, d, H 2& H6), 7.40-7.38 (3H, m, H3, H4, H5), 7.51–7.47 (1H, d, J = 16.0 Hz, -CHα), 7.58–7.56 (2H, d, H3'&H5'), 7.77–7.73 (1H, d, J = 16.0 Hz, -CHβ), 7.96–7.94 (2H, d, H2'&H6'). ESI-MS (*m/z*): Calculated- 288.7918, Observed-288.7915.

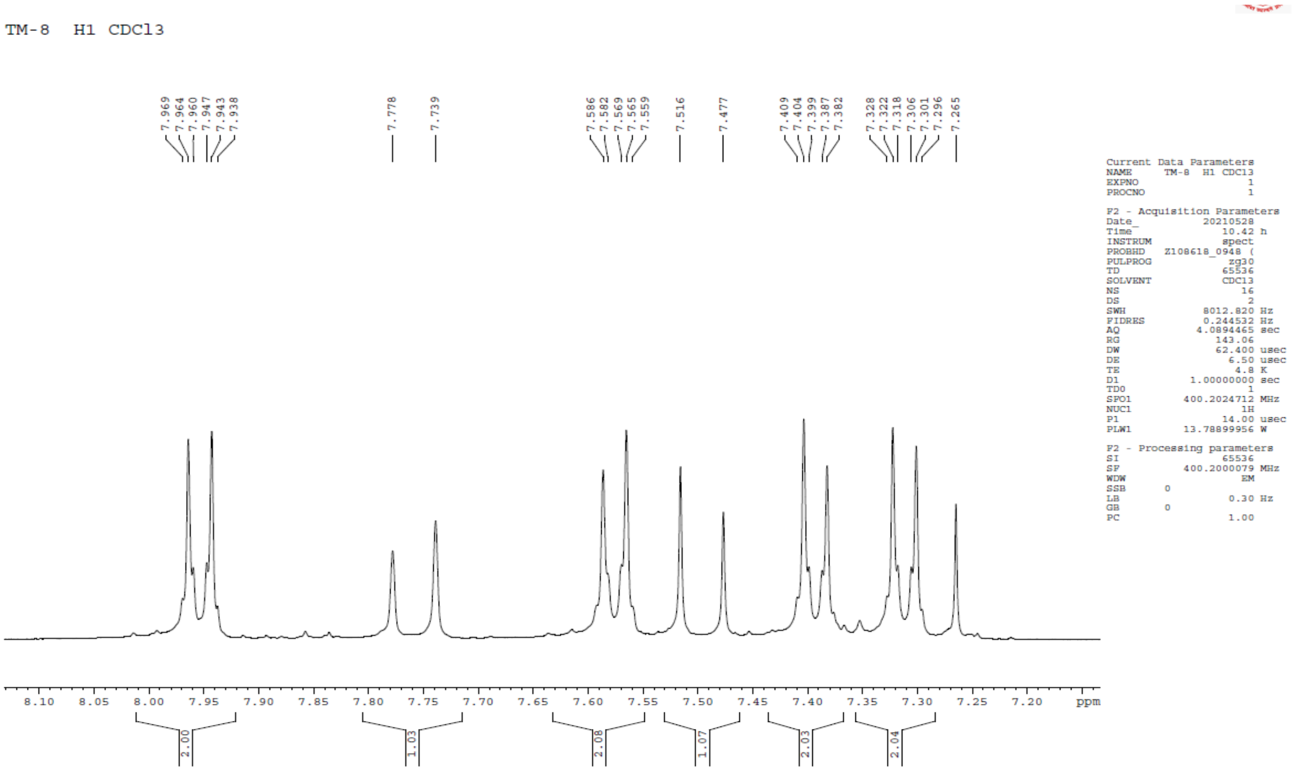

Figure S3. Compound **TM8** ^{1}H-NMR spectrum

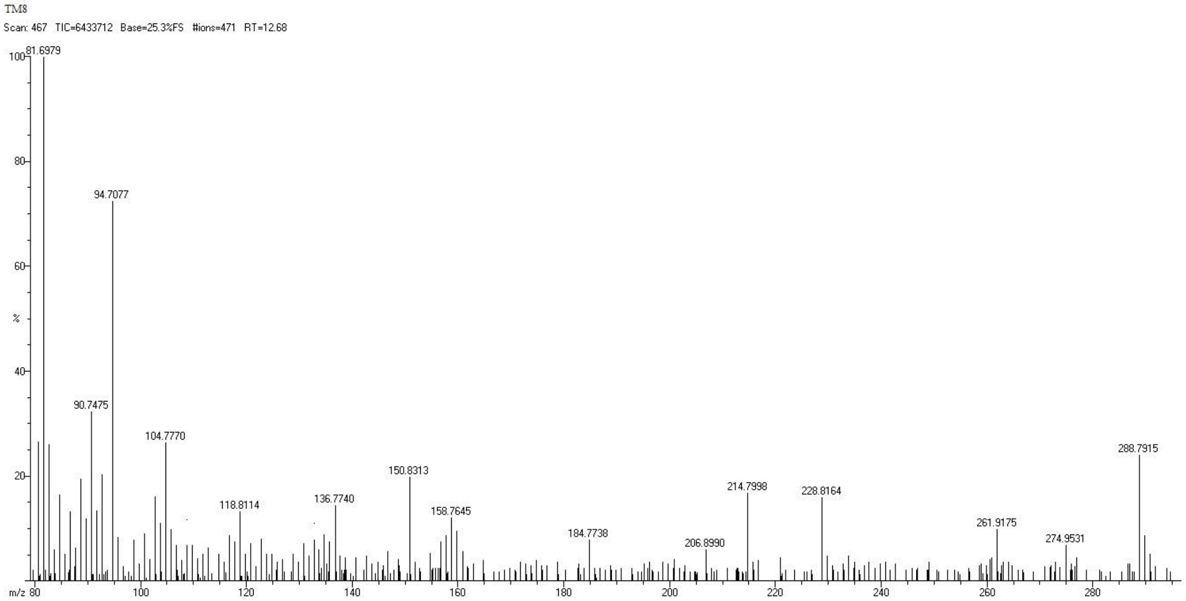

Figure S4. Compound **TM8** ESI MS spectrum

(2*E*)-3-(4-bromophenyl)-1-[4-(methylsulfanyl)phenyl]prop-2-en-1-one (**TM9**): ^{1}H-NMR (400 MHz, $CDCl_3$) δ: 2.54 (3H, s, S-CH_3), 7.32–7.30 (2H, d, H 2& H6), 7.51-7.49 (3H, m, H3, H4, H5), 7.55–7.51 (1H, d, J = 16.0 Hz, -CHα), 7.56–7.54 (2H, d, H3'&H5'), 7.76–7.72 (1H, d, J = 16.0 Hz, -CHβ), 7.96–7.94 (2H, d, H2'&H6'). ESI-MS (*m/z*): Calculated- 333.2428, Observed-333.2425.

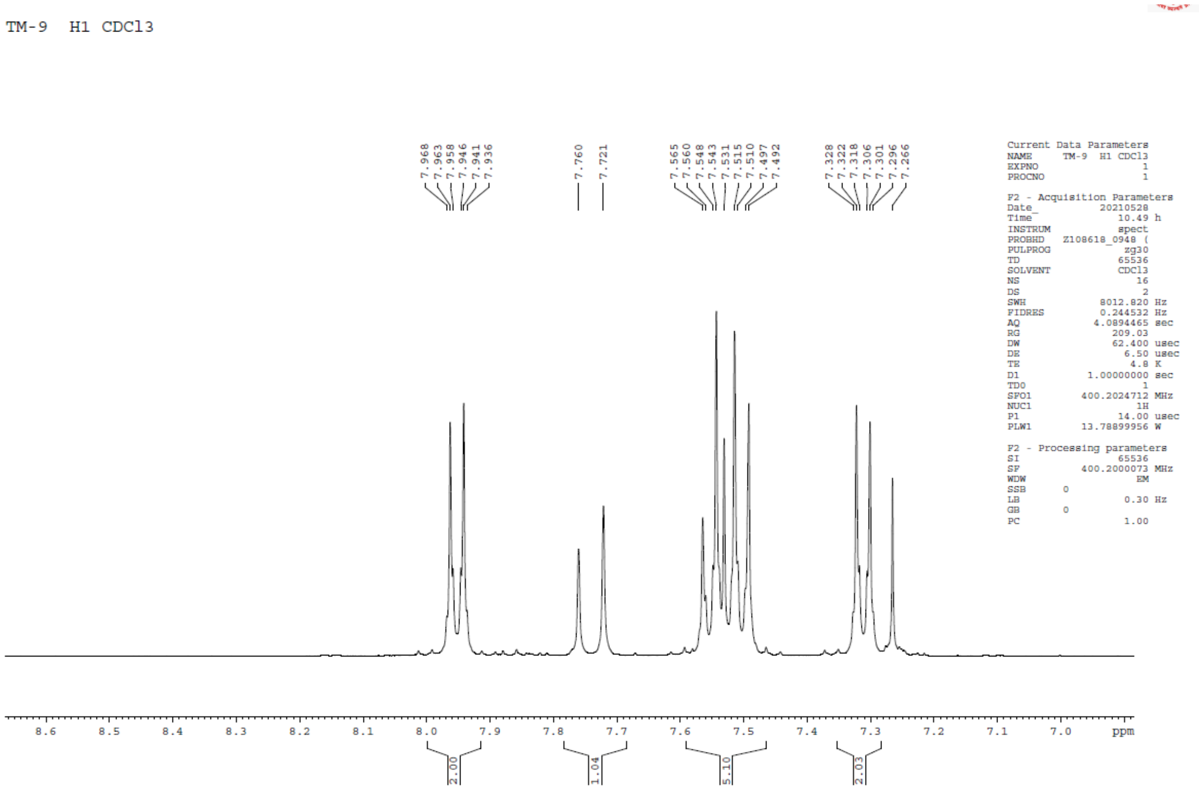

Figure S5. Compound **TM9** ^{1}H-NMR spectrum

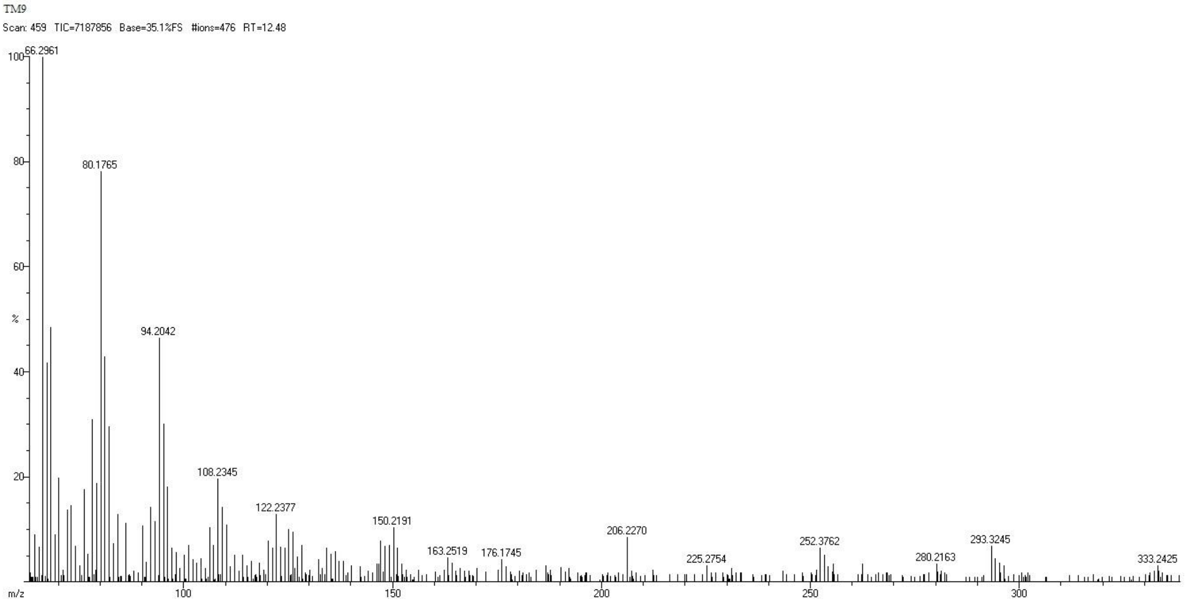

Figure S6. Compound **TM8** ESI MS spectrum

(2*E*)-3-(4-fluorophenyl)-1-[4-(methylsulfanyl)phenyl]prop-2-en-1-one **(TM10)**: ^{1}H-NMR (400 MHz, $CDCl_3$) δ: 2.54 (3H, s, S-CH_3), 7.13–7.11 (2H, d, H 2& H6), 7.32-7.30 (3H, m, H3, H4, H5), 7.47–7.43 (1H, d, J = 16.0 Hz, -CHα), 7.65–7.63 (2H, d, H3′&H5′), 7.79–7.75 (1H, d, J = 16.0 Hz, -CHβ), 7.96–7.94 (2H, d, H2′&H6′). ESI-MS (*m/z*): Calculated- 272.3372, Observed-272.3370.

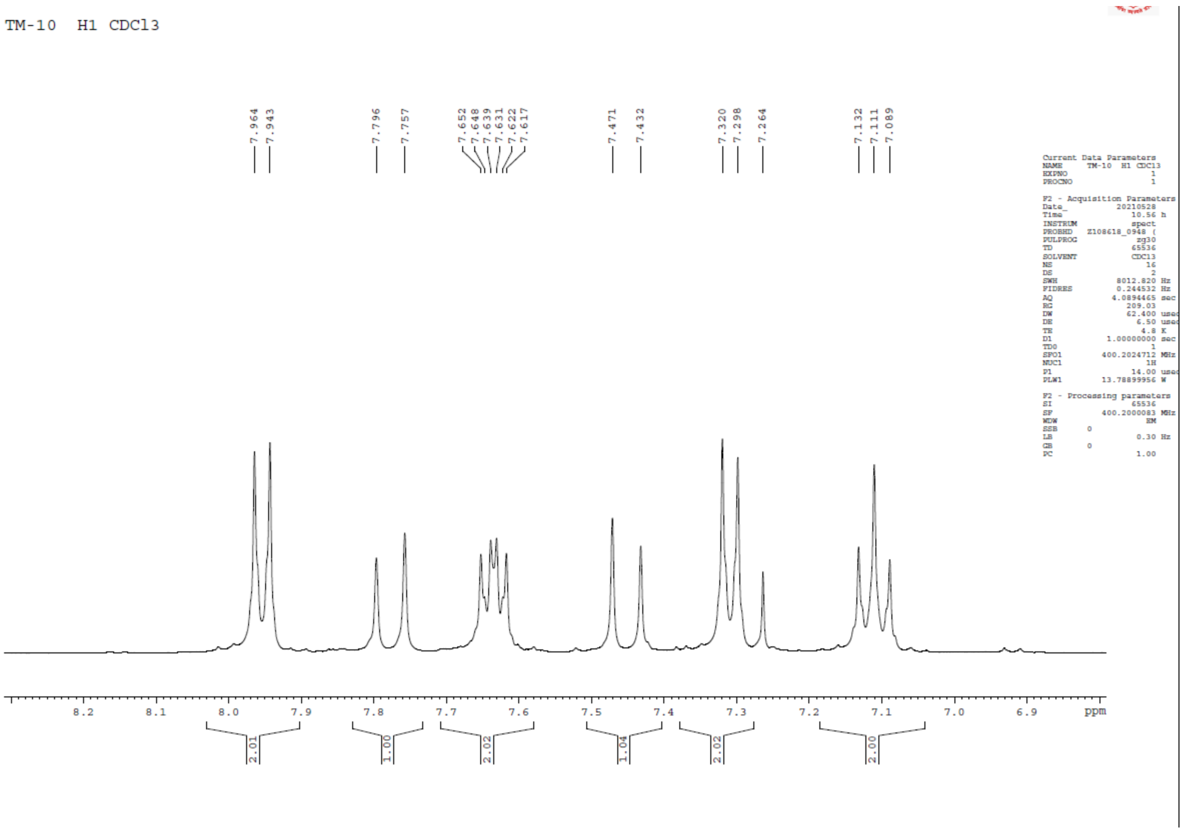

Figure S7. Compound **TM10** ^{1}H-NMR spectrum

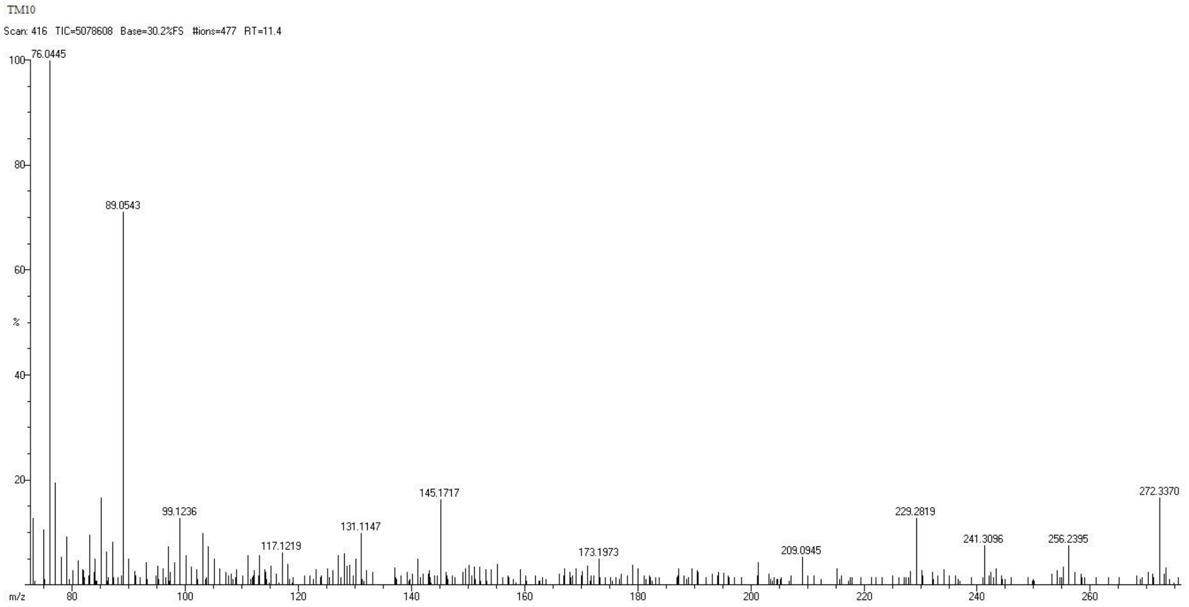

Figure S8. Compound **TM10** ESI MS spectrum
